# Spatial Ability Explains the Male Advantage in Approximate Arithmetic

**DOI:** 10.3389/fpsyg.2016.00306

**Published:** 2016-03-07

**Authors:** Wei Wei, Chuansheng Chen, Xinlin Zhou

**Affiliations:** ^1^Department of Psychology and Behavioral Sciences, Zhejiang UniversityHangzhou, China; ^2^State Key Laboratory of Cognitive Neuroscience and Learning, Siegler Center for Innovative Learning, Beijing Normal UniversityBeijing, China; ^3^Department of Psychology and Social Behavior, University of California, IrvineCA, USA

**Keywords:** gender difference, approximate arithmetic, spatial ability

## Abstract

Previous research has shown that females consistently outperform males in exact arithmetic, perhaps due to the former’s advantage in language processing. Much less is known about gender difference in approximate arithmetic. Given that approximate arithmetic is closely associated with visuospatial processing, which shows a male advantage we hypothesized that males would perform better than females in approximate arithmetic. In two experiments (496 children in Experiment 1 and 554 college students in Experiment 2), we found that males showed better performance in approximate arithmetic, which was accounted for by gender differences in spatial ability.

## Introduction

Gender differences in mathematical performance have been an important area of research because researchers and policy makers alike have been concerned about the under representation of women in mathematics-intensive fields or Science, Technology, Engineer, and Mathematics (STEM; Hyde and Linn, 2006; Halpern et al., 2007; Guiso et al., 2008; Hyde et al., 2008; Ceci et al., 2009; Nosek et al., 2009; Else-Quest et al., 2010; Shen, 2013). Many studies have been conducted to investigate the cognitive, socio-cultural, and biological origins of these differences (Halpern et al., 2007; Kovas et al., 2007; Guiso et al., 2008).

Although male advantage in mathematics has been widely reported, it is by no means the only story in town (Spelke, 2005). For example, Hyde and Linn (2006), Hyde et al. (2008) have emphasized the gender similarity hypothesis. Moreover, there is evidence that at an early age females show better performance in arithmetic than do males (Linn and Hyde, 1989; Wei et al., 2012). One possible explanation of such an advantage is that arithmetic tends to rely on language processing (Dehaene et al., 1999; Lemer et al., 2003), which shows a female advantage (Wei et al., 2012). As Wei et al. (2012) found, after controlling for verbal ability, gender differences in mathematical performance disappeared.

Some arithmetic tasks, however, may not involve much language processing. Distinct from exact arithmetic, approximate arithmetic (e.g., “Of 3 and 8, which number is closer to the answer to the problem 4+5?”) is believed to involve less verbal processing but more number sense and visuospatial processing (Dehaene et al., 1999). Studies have found that approximate arithmetic could be performed without symbols and language (Pica et al., 2004). Young children without formal education can perform large-number symbolic approximate arithmetic (Gilmore et al., 2007). Neuroimaging studies further supported the distinction between exact and approximate arithmetic. It has been found that exact arithmetic relies on the language system, whereas approximate arithmetic relies on the numerical magnitude processing system or the internal “number line” (Dehaene et al., 1999). Specifically, approximate arithmetic recruits the parietal lobe, which is involved in visuo-spatial processing.

In the current study, we recruited two age groups of students to examine gender differences in approximate arithmetic. Given that males show better performance in spatial ability (Voyer et al., 1995) and that spatial ability is linked to approximate arithmetic as mentioned above, we hypothesized that males would outperform females in approximate arithmetic, and that spatial ability would be the cognitive mechanism for the gender difference in approximate arithmetic.

## Experiment 1

### Materials and Methods

#### Participants

Children in 6th—8th grades students were recruited for the study. Children came from two Chinese cities, Liuzhou (Guangxi Province) and Beijing. There were 496 children (234 males and 262 females), 11.0–15.9 years old. All participants were native Chinese speakers and had normal or corrected-to-normal eyesight. This study was approved by the Institute of Cognitive Neuroscience and Learning at Beijing Normal University and the principals of the schools.

#### Procedure

Participants took computerized mathematical and other cognitive tests in a computer room in groups of about 30–40 students per class. They were monitored by 2–3 experimenters and, in the case of 6th–8th grade students, by the class’s teacher as well. Instructions and a practice session were given before each formal test. The tasks were administered in the same order for all students. Participants responded by pressing “P” or “Q” on the keyboard for three of the five tasks (see below), using the mouse for the spatial working memory task, and entering a numerical value for the approximate arithmetic task. Participants’ responses were automatically recorded and sent over the internet to a server located in our laboratory at the university.

#### Tasks

All the tasks were programmed using Web-based applications available at: www.dweipsy.com/lattice (Wei et al., 2012).

##### Symbolic approximate arithmetic

This task was based on Levine’s (1982) *Test of Estimation Ability (TEA)*. The open–ended paradigm (Levine, 1982; Rubenstein, 1985; Dowker, 1992; Dowker et al., 1996) was adopted in the current study to test the ability of approximate arithmetic. An equation was presented in the middle of the screen. On the top the screen, there was a time bar, indicating 15 s. To ensure that the participants could not calculate the exact answer in 15 s, we used multiple digits for all equations (see **Table 1**). Participants were asked to come up with the best approximate answer for the equation in 15 s. Participants entered the answer into an input box at the bottom of the screen. The formal test included 40 trials, including addition, subtraction, multiplication, and division. The four operations were presented randomly for each participant. Both integral and decimal arithmetic was used in this task.

**Table 1:** The test of symbolic approximate arithmetic.

Item/Operation	Addition	Subtraction	Multiplication	Division
1	1752 + 9339	8473 - 1247	581 × 64	6.664 ÷ 0.98
2	8928 + 5397	10395 - 13657	735 × 44	4144 ÷ 37
3	4578 + 3566	27534 - 11846	23 × 76	23596 ÷ 68
4	8546 + 5773	7814 - 1937	397 × 35	11515 ÷ 47
5	3696 + 1276	57631 - 14768	34 × 87	16068 ÷ 78
6	23.27 + 594.9	93.12 - 148.73	93 × 0.24	5.472 ÷ 57
7	749.6 + 4737.9	574.21 - 18.796	7.2 × 98.6	2352 ÷ 24
8	6.759 + 0.2867	5.614 - 10.4935	0.893 × 3.7	403.76 ÷ 0.98
9	926.4 + 75.72	208.3 - 129.26	2.17 × 0.83	66.3 ÷ 6.5
10	38.69 + 629.8	15.94 - 10.798	0.68 × 7.9	343.2 ÷ 22


##### Three-dimensional mental rotation

This task was based on Shepard’s mental rotation task (Shepard and Metzler, 1971). For each trial, one three-dimensional image was presented on the upper part of the screen, and two others on the lower part. Participants were asked to choose one from the bottom to match with the top; the matching image could be identified only by mental rotation. Participants were asked to press the “Q” key if he/she chose the image on the left, or the “P” key he/she chose the image on the right. The formal test included 180 trials and was limited to 3 min. The rotation angles of the images were 15°, 30°, …, 345°, with a step of 15°. Each trial would remain on the screen until participants responded by pressing “P” or “Q”.

##### Raven’s progressive matrices

The Raven’s Progressive Matrices test (Raven, 1998) was used to assess general intelligence. In this test, participants needed to identify the missing segment of a figure according to the figure’s inherent regularity. They should press “Q” if the missing segment was on the left or “P” if it appeared on the right. The formal test included 80 trials and was limited to 4 min.

##### Spatial working memory

This task was similar to Corsi block task (Corsi, unpublished doctoral dissertation). Non-overlapping dots were sequentially presented in an implicit lattice of 3 × 3 on the computer screen. Each dot was presented for 1 s, and dots were presented with an interval of 1 s. After the last dot was presented and disappeared, a cue would be presented on the screen to ask the participants to click the positions where the dots had appeared in the same sequence as their appearance. The number of dots ranged from 3 to 7. There was no feedback to participants. The average distance between the position where the dot appeared and the position where participants clicked was calculated and treated as an index of spatial working memory.

##### Word semantic processing

The format of this task was similar to the one used by Siegel and Ryan (1988) and So and Siegel (1997). Materials in the task were adapted from the language examinations used in China in recent years. In the task, a sentence was presented in the center of the computer screen with a word missing. Participants needed to select one of two candidate words presented beneath the sentence by pressing a left or a right key. The stimulus remained on the screen until the participants responded. The formal test included 120 trials and was limited to 5 min.

For each of the time-limited tasks (i.e., mental rotation, Raven’s Progressive Matrices, and word semantic processing), we calculated scores using Guilford formula (Guilford proposed a correction formula “*S* = *R* – *W*/(*n* – 1)” (S: the adjusted number of items that the participants can actually perform without the aid of chance. R: the number of correct responses, W: the number of incorrect responses. n: the number of alternative responses to each item; Guilford, 1936). For the spatial working memory task, as mentioned earlier, the average distance between the position where the dot appeared and the position where participants clicked was calculated. We then subtracted the average distance from 200 to create a score for spatial working memory. For the approximate arithmetic task, we used the formula “100 – |(PR – EA)/(PR + EA)|× 100” to calculate accuracy in approximate arithmetic. PR refers to participant’s response and EA the exact answer. Using this formula, accuracy scores in approximate arithmetic would have the theoretical range from 0 to 100.

#### Data Analysis

Because our sample came from 20 classes, it was necessary to first investigate whether the nested data needed to be analyzed with multilevel models. We used the unconditional means model to compute the intraclass correlation coefficients (ICC) (Peugh and Enders, 2005). The ICC was 0.12 for approximate arithmetic, suggesting significant variability at the between-classroom level. Therefore, we conducted multilevel models by using the MIXED procedure in SPSS for all data analyses. The following equations were used:

$$Level\,1\,:{Score}_{ij}=\beta_{0j}+\beta_{1j}({Age}_{ij})+\beta_{2j}({Gender}_{ij})+\beta_{3j}({Co\,\mathrm{var}iates}_{ij})+\gamma_{ij}$$

Where Score*_ij_* was the score of approximate arithmetic for participant *i* in class *j*, and β_0_*_j_* was the mean score for class *j.* β_1_*_j_*, β_2_*_j_*, and β_3_*_j_* were the slopes of age, gender and covariates (i.e., scores of various tests) predicting the score within class *j*. γ*_ij_* was the random component of the score for participant *i* in class *j*.

Level⁢2:β0j=γ00+γ01⁢Regionij+μ0jβ1j=γ10β2j=γ20β3j=γ30

Where β_0_*_j_* was the mean score for class *j*, γ_00_ was the grand mean score across all classes, γ_01_ was the slope of level-2 variable region predicting the mean score for class *j*, and μ_0_*_j_* was the random component of the mean score for class *j*. β_1_*_j_*, β_2_*_j_*, and β_3_*_j_*, were the slopes of age, gender and covariates predicting the mean score for class *j*.

Combined:

Scoreij=γ00+γ01(Region)+γ10(Age)+γ20(Gender)+γ30(Co⁢variates)+μ0j+γij

#### Results and Discussion

Of the 19840 answers (496 children × 40 trials), 210 (1.1%) were correct exact answers. **Table 2** shows the mean scores and standard deviations of all tasks. **Table 3** shows the inter-task correlations. All correlations were significant.

**Table 2:** Means, standard deviations, and gender differences for all tasks (Experiment 1).

Task	Younger children (ages 11–12)				Older children (13–15)				Tests of gender difference (multilevel model)	
			
	Beijing		Liuzhou		Beijing		Liuzhou			
						
	Boys	Girls	Boys	Girls	Boys	Girls	Boys	Girls	*b*	*t*
Approximate arithmetic	62.2 (20.1)	55.7 (17.6)	53.3 (23.8)	51.0 (17.9)	62.6 (15.2)	60.4 (13.3)	55.3 (22.8)	54.1 (16.4)	-3.59	*t* (479.57) = -2.22ˆ*
Mental rotation	21.3 (8.4)	18.3 (9.7)	23.3 (8.0)	19.7 (9.1)	21.9 (9.2)	20.2 (10.7)	23.0 (8.4)	18.9 (8.1)	-3.48	*t* (481.51) = -4.36ˆ***
Word semantic processing	27.8 (7.4)	31.3 (7.9)	27.6 (7.9)	29.3 (7.2)	29.1 (7.1)	33.2 (6.4)	27.3 (7.5)	31.2 (6.4)	3.33	*t* (483.42) = 5.21ˆ***
Spatial working memory	153.5 (20.5)	154.3 (15.8)	151.2 (24.3)	149.8 (22.3)	158.2 (12.4)	152.1 (19.2)	148.5 (27.7)	149.1 (28.4)	1.49	*t* (482.46) = 0.74
Raven’s Progressive Matrices	19.9 (7.1)	19.8 (6.2)	20.2 (7.6)	21.7 (5.9)	18.8 (6.2)	20.9 (5.9)	19.7 (4.7)	21.3 (4.5)	1.10	*t* (479.32) = 2.14ˆ*


*^∗^*p* < 0.05; ^∗∗∗^*p* < 0.001; Gender was coded to 0 for boys and 1 for girls.*

**Table 3:** Correlations among all tasks (Experiment 1).

Tasks		1	2	3	4
1	Approximate arithmetic	-			
2	Mental rotation	0.16ˆ**	-		
3	Word semantic processing	0.21ˆ***	0.12ˆ**	-	
4	Spatial working memory	0.31ˆ***	0.20ˆ***	0.16ˆ***	-
5	Raven’s Progressive Matrices	0.14ˆ**	0.20ˆ***	0.12ˆ**	0.13ˆ**


*^∗∗^*p* < 0.01; ^∗∗∗^*p* < 0.001.*

According to multilevel model analysis, boys outperformed girls in approximate arithmetic and mental rotation, whereas girls outperformed boys in word semantic processing and Raven’s Progressive Matrices. There was no gender difference in spatial working memory (**Table 2**).

The analysis showed no differences between older and younger children for all tasks [*b* = –1.13, *t*(330) = –0.54, *p* = 0.586 for symbolic approximate arithmetic; *b* = 0.57, *t*(168) = 0.60, *p* = 0.551 for mental rotation; *b* = –0.94, *t*(222) = –1.21, *p* = 0.227 for word semantic processing; *b* = 1.17, *t*(249) = 0.47, *p* = 0.638 for spatial working memory; *b* = 0.95, *t*(216) = 1.51, *p* = 0.133 for Raven’s Progressive Matrices]. In the multilevel model (when classroom effect was considered), no region differences were found for all tasks [*b* = –5.49, *t*(18) = –1.61, *p* = 0.125 for symbolic approximate arithmetic; *b* = 0.96, *t*(16) = 0.81, *p* = 0.429 for mental rotation; *b* = –1.42, *t*(21) = –1.41, *p* = 0.172 for word semantic processing; *b* = –4.73, *t*(20) = –1.38, *p* = 0.181 for spatial working memory; *b* = 1.10, *t*(15) = 1.25, *p* = 0.229 for Raven’s Progressive Matrices]. None of the interactions involving gender and approximate arithmetic were significant [*b* = 0.12, *t*(477) = 0.03, *p* = 0.979 for gender × region; *b* = 5.26, *t*(479) = 1.06, *p* = 0.288 for gender × age; *b* = –5.37, *t*(476) = –0.079, *p* = 0.431 for gender × region × age].

**FIGURE 1:**
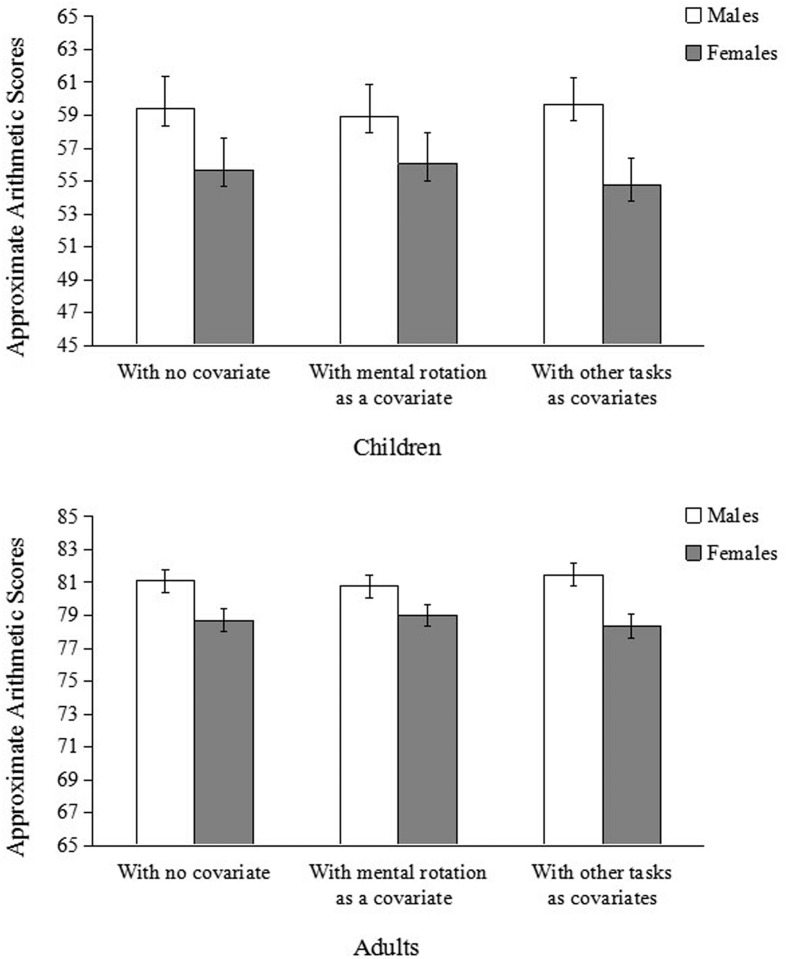
**Average scores in approximate arithmetic of children in Experiment 1 (top) and adults in Experiment 2 (bottom).** The bars on the left show the means without controlling for covariate; the bars in the middle show the adjusted means after controlling for performance on the mental rotation task only; and the bars on the right show the adjusted means after controlling for performance on all other tasks except mental rotation task. Error bars indicate standard errors.

Multilevel model analysis showed that after controlling for mental rotation, gender difference in approximate arithmetic disappeared (**Table 4** and **Figure 1**). After controlling for any one of the other measures, however, gender difference in approximate arithmetic still remained (**Table 4**). Even after controlling for all the other measures simultaneously, gender difference in approximate arithmetic remained (**Figure 1**), *b* = –4.74, *t*(476.27) = –2.97, *p* = 0.003.

**Table 4:** Results from multilevel modeling showing gender differences in approximate arithmetic (Experiment 1).

Covariate	Approximate arithmetic		
	
	*b*	*SE × b*	*t*
None	–3.59	1.61	*t*(479.57) = –2.22^∗^
Mental rotation	–2.73	1.64	*t*(481.02) = –1.66
Word semantic processing	–5.02	1.64	*t*(477.95) = –3.06^∗∗^
Spatial working memory	–3.21	1.57	*t*(479.47) = –2.05^∗^
Raven’s Progressive Matrices	–4.02	1.61	*t*(478.10) = –2.50^∗^


*^∗^*p* < 0.05; ^∗∗^*p* < 0.01; Gender was coded as 0 for boys and 1 for girls. SE: standard error.*

We further examined whether mental rotation could explain the gender differences in other tasks. The results showed that these gender differences could not be explained by mental rotation: including gender difference in word semantic processing, *b* = 3.76, *t*(485.73) = 5.82, *p* < 0.0001; and in Raven’s Progressive Matrices: *b* = 1.57, *t*(482.95) = 3.05, *p* = 0.002.

**FIGURE 2:**
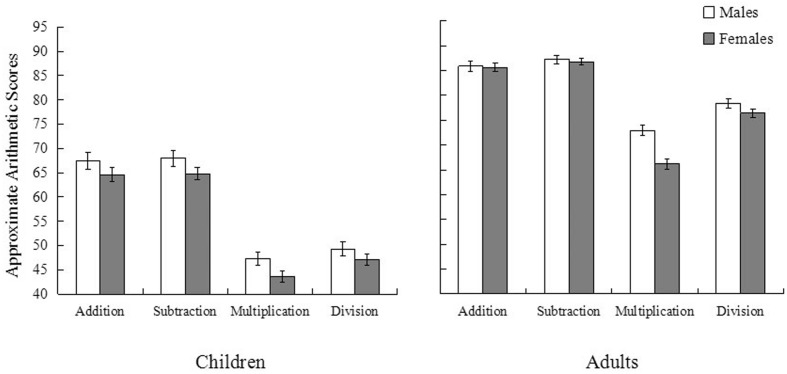
**Mean scores of approximate arithmetic (addition, subtraction, multiplication, and division) of children in Experiment 1 (left) and adults in Experiment 2 (right).** Error bars indicate standard errors.

To further examine whether gender differences were consistent across the four arithmetic operations (i.e., addition, subtraction, multiplication, and division), we re-conducted the multilevel model analysis, with arithmetic operation as a within-subject variable and gender, age, and region as between-subject variables. Results showed that gender, age, and region had significant main effects, *b* = –2.80, *t*(1883) = –2.62, *p* = 0.009 for gender, *b* = 2.70, *t*(1883) = 2.41, *p* = 0.016 for age, and *b* = 6.65, *t*(1883) = 6.03, *p* < 0.001 for region. No significant interaction effects were found among the variables, *b* = 0.11, *t*(1883) = 0.10, *p* = 0.920. That is, boys outperformed girls for each operation (**Figure 2**). Controlling for scores on the mental rotation task, gender difference in approximate arithmetic was no longer significant, *b* = –1.71, *t*(1871) = –1.59, *p* = 0.113.

The current investigation focused on children from primary and secondary schools in two regions of China. As expected, boys performed better than girls on approximate arithmetic. When we controlled for the three-dimensional mental rotation task, gender difference in approximate arithmetic disappeared. However, after controlling for the other cognitive tasks, gender difference in approximate arithmetic remained.

To our knowledge, little research has been conducted to explore the development of gender difference in arithmetic. Thus, the second experiment was conducted to investigate whether the gender differences in approximate arithmetic would exist in adults, and whether the same cognitive mechanisms would explain such gender differences.

## Experiment 2

### Materials and Methods

#### Participants

The adult sample of 554 college students (250 males and 304 females, 18.0–21.9 years old) was recruited from Harbin Normal University and Southwest University. It included 292 students majoring in sciences such as chemistry, computer science, biology, mathematics, and physics, and the others majoring in arts and humanities such as Chinese literature, education, history, and political science. All participants were native Chinese speakers and had normal or corrected-to-normal eyesight. They gave written consent form after procedure was fully explained. They received 30 RMB (about US$ 4.8) as a compensation for their time.

#### Procedure and Tasks

The procedure and tasks were the same as in Experiment 1.

#### Data Analysis

Similar multilevel models as in Experiment 1 were used in the current data analysis. The main equation was as follows:

$${Score}_{ij}=\gamma_{00}+\gamma_{01}\left(Major\right)+\gamma_{10}(Gender)+\gamma_{20}\left(Co\,\mathrm{var}iates\right)+\mu_{0j}+\gamma_{ij}$$

#### Results and Discussion

Ten participants (seven males and three females) were deleted as outliers because their approximate arithmetic scores were 3 SD above or below the group mean. Of the remaining 21760 responses (544 participants × 40 trials), 1481 responses (6.8%) were exact answers. **Table 5** shows the mean scores and standard deviations of all tasks. **Table 6** shows the inter-task correlations. All correlations were significant.

**Table 5:** Means, standard deviations, and gender differences for all tasks (Experiment 2).

Tasks	Arts		Science		Gender difference
			
	Males	Females	Males	Females	*F*
Approximate arithmetic	79.9 (12.8)	77.5 (10.2)	81.9 (12.0)	80.2 (9.7)	-2.11ˆ*
Mental rotation	27.4 (8.0)	24.5 (8.4)	28.9 (9.6)	26.3 (7.9)	-3.71ˆ***
Word semantic processing	36.2 (8.6)	40.6 (6.4)	36.0 (6.5)	38.3 (6.3)	4.95ˆ***
Spatial working memory	149.5 (27.5)	153.8 (18.7)	154.1 (22.6)	154.5 (20.2)	1.16
Raven’s Progressive Matrices	23.1 (7.4)	23.3 (7.0)	22.8 (6.6)	23.8 (5.7)	0.94


*^∗^*p* < 0.05; ^∗∗∗^*p* < 0.001.*

**Table 6:** Correlations among all tasks (Experiment 2).

Tasks		1	2	3	4
1	Approximate arithmetic	-			
2	Mental rotation	0.17ˆ***	-		
3	Word semantic processing	0.10ˆ*	0.12ˆ**	-	
4	Spatial working memory	0.18ˆ***	0.22ˆ***	0.13ˆ**	-
5	Raven’s Progressive Matrices	0.22ˆ***	0.25ˆ***	0.24ˆ***	0.26ˆ***


*^∗^*p* < 0.05; ^∗∗^*p* < 0.01; ^∗∗∗^*p* < 0.001.*

Males outperformed females in approximate arithmetic and mental rotation, whereas females outperformed males in word semantic processing. There was no gender difference in spatial working memory and Raven’s Progressive Matrices. Science students were superior to arts students in approximate arithmetic, *b* = 2.43, *t*(551) = 2.55, *p* = 0.011. No difference across majors was found for other tasks: Raven’s Progressive Matrices, *b* = 0.52, *t*(6.48) = 0.59, *p* = 0.577; spatial working memory, *b* = 2.40, *t*(4.51) = 1.21, *p* = 0.286; word semantic processing, *b* = –0.86, *t*(6.36) = –0.63, *p* = 0.550. The interaction between gender and major was not significant [*b* = –0.70, *t*(550) = –0.37, *p* = 0.715].

Results showed that after controlling for mental rotation, gender difference in approximate arithmetic disappeared (**Table 7** and **Figure 1**). But after controlling for other tasks, gender difference in approximate arithmetic remained (**Table 7** and **Figure 1**). We further examined whether mental rotation could explain gender differences in performance on other tasks. The results showed that gender differences in word semantic processing could not be explained by mental rotation, *b* = –3.32, *t*(549.87) = –5.62, *p* < 0.001.

**Table 7:** Results from multilevel modeling showing gender differences in approximate arithmetic (Experiment 2).

Covariate	Approximate arithmetic		
	
	*b*	*SE × b*	*t*
None	–2.01	0.95	*t*(551) = –2.11^∗^
Mental rotation	–1.50	0.96	*t*(550) = –1.57
Word semantic processing	–2.78	0.97	*t*(550) = –2.87^∗∗^
Spatial working memory	–2.22	0.94	*t*(550) = –2.37^∗^
Raven’s Progressive Matrices	–2.26	0.93	*t*(550) = –2.43^∗^


*^∗^*p* < 0.05; ^∗∗^*p* < 0.01; Gender was coded as 0 for boys and 1 for girls.*

To examine whether other cognitive tasks except for mental rotation could explain gender difference in approximate arithmetic, we controlled for spatial working memory, word semantic processing, and Raven’ Progressive Matrices simultaneously. Gender difference remained, *b* = 2.81, *t*(548) = 2.98, *p* = 0.003.

To further examine whether gender differences were consistent across the four arithmetic operations (i.e., addition, subtraction, multiplication, and division), we conducted repeated measure ANOVA with gender and students’ major as between-subject variables. Results showed that gender [*b* = 3.01, *t*(2006) = 2.19, *p* = 0.029] and major [*b* = 4.02, *t*(2006) = 2.86, *p* = 0.004] had significant main effects but no significant interaction [*b* = 0.72, *t*(2006) = 1.15, *p* = 0.250]. Males outperformed females, and science students outperformed arts and humanities students on each operation. Results showed that after controlling for scores on the mental rotation task, gender differences in approximate arithmetic disappeared [*b* = 1.08, *t*(2008) = 1.51, *p* = 0.132].

In Experiment 2, we found similar results as those found with children in Experiment 1. Males performed better than females in approximate arithmetic. Controlling for the mental rotation task, gender difference disappeared; but controlling for the other cognitive tasks, males still had an advantage over females.

## Discussion

The goal of the current study was to examine gender differences in approximate arithmetic. Our results showed that males performed better in approximate arithmetic than did females, and this gender difference disappeared after controlling for spatial ability.

### Cognitive Mechanism of Approximate Arithmetic

Approximate arithmetic has a high correlation with spatial ability. Behavioral studies showed that participants represented numerical magnitude on the mental number line in the symbolic and non-symbolic approximate arithmetic tasks (McCrink and Wynn, 2004; Knops et al., 2009) and that the mental number line has a spatial property (Dehaene et al., 1993). Neuroimaging studies have shown that approximate arithmetic and spatial processing share a similar brain basis, typically involving the parietal cortex (Dehaene et al., 1999; Stanescu-Cosson et al., 2000; Lemer et al., 2003). Compared to the non-mathematician control group, mathematicians excelled in approximate arithmetic (Dowker, 1992; Dowker et al., 1996) and their parietal cortex (a brain region involved in spatial processing, (Corbetta et al., 1998; Kosslyn et al., 1998; Gitelman et al., 1999; Zacks et al., 1999) showed greater gray matter density (Aydin et al., 2007).

Approximate arithmetic relies on spatial ability, but not on language ability. In a study of language and approximate arithmetic (Spelke and Tsivkin, 2001), bilingual students were trained to perform exact and approximate arithmetic problems in two languages. Results showed that, for exact arithmetic, the language used for training mattered, but for approximate arithmetic, the language used for training did not matter. A recent study also found that children with language impairment had lower accuracy in exact arithmetic, but they had similar performance in approximate arithmetic as compared to the normal children (Nys et al., 2013). From a developmental perspective, approximate arithmetic precedes exact arithmetic because the latter relies on number symbols as language processing. For example, preschool children can perform approximate arithmetic but not exact arithmetic with the same numbers (Gilmore et al., 2007). Similarly, Amazonian indigenes can perform approximate arithmetic, but not exact arithmetic, due to their lack of a formal language-based number system (Pica et al., 2004).

### Gender Difference in Spatial Ability

Many studies have shown that males outperform females on spatial ability tasks, especially the mental rotation tasks (Voyer et al., 1995). Gender difference in spatial ability emerges as early as about 3–5 months of age (Moore and Johnson, 2008; Quinn and Liben, 2008) and is evident to the age of 95 years (De Frias et al., 2006; Tran and Formann, 2008). Moreover, based on data from more than 200,000 subjects from 53 nations, Lippa et al. (2010) showed that males performed better than females on visuospatial tasks.

Neuroimaging studies have showed that males have a larger parietal lobule (Frederikse et al., 1999), which could explain males’ superiority in spatial ability (Koscik et al., 2009). The right parietal cortex is involved in visuospatial processing during arithmetic tasks (see Arsalidou and Taylor, 2011, for a meta-analysis). For example, when the right parietal cortex was suppressed, participants could not perform spatial tasks (Bjoertomt et al., 2002; Rosenthal et al., 2009). Interestingly, when males perform the spatial tasks, their bilateral hemispheres are involved, whereas females tend to rely on their right hemisphere (Gur et al., 2000; Clements et al., 2006). Taken together, it is plausible that males’ larger parietal cortex (especially in the right hemisphere, Caviness et al., 1996; Baibakov and Fedorov, 2010) accounts for their better performance on spatial tasks (Moore and Johnson, 2008; Quinn and Liben, 2008).

In sum, our study showed consistent gender differences in approximate arithmetic favoring males across age groups and identified gender differences in spatial ability as a potential cognitive mechanism. These results have important implications for later development of mathematical cognition. Future research should pay more attention to the understudied approximate arithmetic, which may be important for advanced mathematics.

## Author Contributions

WW: designed the experiment, collected and analyzed the data and drafted the manuscript; CC: revised the manuscript; XZ: designed the experiment, revised the manuscript.

## Conflict of Interest Statement

The authors declare that the research was conducted in the absence of any commercial or financial relationships that could be construed as a potential conflict of interest.
